# OSA Patient Monitoring Based on the Beidou System

**DOI:** 10.3389/fpubh.2021.745524

**Published:** 2021-11-16

**Authors:** Cai Liangming, Cai Xiaoqiong, Du Min, Miao Binxin, Lin Minfen, Zeng Zhicheng, Li Shumin, Ruan Yuxin, Hu Qiaolin, Yang Shuqin

**Affiliations:** ^1^School of Physics and Information Engineering, Zhicheng College, Fuzhou University, Fuzhou, China; ^2^Third Institute of Oceanography, State Oceanic Administration, Xiamen, China; ^3^Laboratory of Eco-Industrial Green Technology Wuyi College, Wuyishan, China; ^4^College of Zhicheng, Fuzhou University, Fuzhou, China

**Keywords:** IoT–internet of things, OSA patient rescue system, Beidou indicator, STM32 microcontroller implementation, android

## Abstract

This paper presents an OSA patient interactive monitoring system based on the Beidou system. This system allows OSA patients to get timely rescue when they become sleepy outside. Because the Beidou position marker has an interactive function, it can reduce the anxiety of the patient while waiting for the rescue. At the same time, if a friend helps the OSA patients to call the doctor, the friend can also report the patient's condition in time. This system uses the popular IoT framework. At the bottom is the data acquisition layer, which uses wearable sensors to collect vital signs from patients, with a focus on ECG and SpO2 signals. The middle layer is the network layer that transmits the collected physiological signals to the Beidou indicator using the Bluetooth Low Energy (BLE) protocol. The top layer is the application layer, and the application layer uses the mature rescue interactive platform of Beidou. The Beidou system was developed by China itself, the main coverage of the satellite is in Asia, and is equipped with a high-density ground-based augmentation system. Therefore, the Beidou model improves the positioning accuracy and is equipped with a special communication satellite, which increases the short message interaction function. Therefore, patients can report disease progression in time while waiting for a rescue. After our simulation test, the effectiveness of the OSA patient rescue monitoring system based on the Beidou system and the positioning accuracy of OSA patients have been greatly improved. Especially when OSA patients work outdoors, the cell phone base station signal coverage is relatively weak. The satellite signal is well-covered, plus the SMS function of the Beidou indicator. Therefore, the system can be used to provide timely patient progress and provide data support for the medical rescue team to provide a more accurate rescue plan. After a comparative trial, the rescue rate of OSA patients using the detection device of this system was increased by 15 percentage points compared with the rescue rate using only GPS satellite phones.

## Introduction

Sleep respiratory diseases include obstructive sleep apnea (OSA) and central nervous system sleep apnea (CSA), etc., the research shows that 80% of the patients with sleep apnea syndrome have snoring phenomena (1). Long term sleep apnea can cause dysfunction of the body's system and multiple diseases, such as increasing the risk of obesity and heart disease, and even sudden death (2). According to the World Health Group, the high-risk groups of sleep respiratory diseases mainly involve obese patients and the elderly (3). In middle-aged men and women in the United States, OSA incidence is high, the number of patients with sleep disordered breathing in the world is a large group, and nearly 80–90% of them have apnea syndrome but they are often undiagnosed (4). Therefore, the monitoring and auxiliary regulation of sleep disordered breathing are of great significance for the prevention and diagnosis of sleep and related diseases. One method is to help patients achieve a normal Body Mass Index (BMI), but the patients cannot always return to normal because their perseverance is insufficient, so the method cannot achieve the goal of reducing sleep disorders (SD) (5).

There are ~5,000 standard sleep laboratories in the world, and most sleep laboratories use polysomnography (PSG) as the gold standard for detecting sleep disorders (6). PSG can only be used in specialized clinics or hospitals under constant medical supervision to collect physiological signals from patients with sleep disorders, and then use professional sleep experts to diagnose patients who have sleep disorders based on physiological signals detected by PSG (7). This means that patients must go to specialized medical institutions, which will inevitably increase the burden on patients. Moreover, PSG requires the integration of many sensors on the human body, which is considered invasive, so PSG screening can interfere with sleep (8). In addition, PSG is costly and time consuming, because the PSG test needs professional sleep experts. Therefore, it would be difficult for PSG to become a method for long-term monitoring of patients with sleep disorders (9). Limited data show that patients with severe sleep apnea who do not receive effective treatment will increase the risk of sudden cardiac death. If there is no heart disease, only sleep apnea, the possibility of death is very small. Long-term sleep apnea, if not well-treated, will increase the incidence of a series of cardiovascular diseases including hypertension, pulmonary hypertension, coronary heart disease, heart failure, etc., and will also make the therapeutic effect of these diseases worse. For example, about 70% of refractory hypertension patients with poor blood pressure control have sleep apnea. Therefore, screening sleep apnea is very important for hypertension patients whose blood pressure is difficult to control. Sleep apnea may also increase the incidence of diabetes, fatty liver, stroke, and depression. It can make people sleepy and lack attention during the day, which also increases the possibility of accidents such as traffic accidents or the operation of other dangerous equipment.

Due to problems such as OSA not being effectively solved by surgery, and sleep correction using CPAP causing discomfort to patients with sleep disorders, the data suggests that 80% of the patients with sleep disorders have not been effectively diagnosed or treated in time. There is therefore a need for an Internet of Things (IoT) framework system to monitor OSA patients. This system enables OSA patients to be rescued in time when they are drowsy outdoors. Because the Beidou position marker has an interactive function, it can reduce the anxiety of the patient while waiting for rescue. At the same time, if a friend helps an OSA patient to call a doctor, the friend can also report the patient's condition in time.

The system uses a popular IoT framework. At the bottom is the data acquisition layer, which uses wearable sensors to collect vital signs from patients, with a focus on ECG and SpO2 signals. The middle layer transmits the collected physiological signals to the network layer of the Beidou indicator using the Bluetooth Low Energy (BLE) protocol. The top layer is the application layer, and the application layer uses the mature Beidou rescue interactive platform. Since the GPS indicator does not include a communication satellite, it does not have an SMS function. OSA patients can only passively wait for rescue. In addition, due to the lack of satellites in Asia and the insufficient density of ground-enhanced systems, OSA patients have large positioning errors. The Beidou system is developed by China itself, the main coverage of the satellite is in Asia, and is equipped with a high-density ground enhancement system. Therefore, the Beidou model improves the positioning accuracy and is equipped with a special communication satellite, which increases the short message interaction function. Therefore, patients can report disease progression in a timely manner while waiting for a rescue.

After our simulation test, the effectiveness of the OSA patient rescue monitoring system based on the Internet of Things framework and the positioning accuracy of OSA patients have been greatly improved. Especially when OSA patients work outdoors, the signal coverage of cell phone base stations is relatively weak. The satellite signal is well-covered, plus the SMS function of the Beidou indicator. As a result, the system can be used to provide timely patient progression and provide data support to the medical rescue team to provide a more accurate rescue plan. After a comparative test, the rescue rate of OSA patients using the system's detection equipment increased by 15 percentage points compared to the rescue rate using only GPS satellite phones.

The rest of this paper is structured as follows. Part 2 presents the latest literature on this area of research. Part 3 presents the methods and simulation results of a sleep disorder monitoring system based on the *Beidou system*. Section Experimental study summarizes and suggests the direction for future improvements.

## Related Work

At present, the golden standard for the diagnosis of sleep apnea is polysomnography (PSG) (10). However, PSG is expensive, so it is necessary for special sleep respiratory monitors to connect electroencephalogram (EEG), eye movement, EMG, and other wires to the body surface of patients, so their application is limited to a certain extent (11). In order to reduce the measured physiological signals, the detection method of sleep dyspnea based on SpO2, ECG (Electrocardiogram) signal has become a hot research topic (12).

In the research of detecting sleep dyspnea based on ECG signal, the methods of OSA detection of ECG signal were studied by the University of Wisconsin, Taiwan Jiaotong University and Peking University (13–18). At present, there are several CFDA certified devices in China to diagnose sleep dyspnea by sticking electrodes in the human body (19). The latest research shows that by monitoring the ECG signal, sending it to the neural network through frequency domain and time domain analysis, and establishing a classification model through statistical analysis of the apnea-hypopnea index (AHI), the automatic detection of SDB can be realized to a certain extent (20). However, ECG signal acquisition requires patients to wear at least two ECG electrodes for dynamic monitoring for a long time, which brings serious physical and mental burden to patients. Therefore, finding a physiological signal that can replace ECG and is easier to obtain has become a research direction. Like the ECG signal, there is a slight difference in the interval between consecutive pulse waves. Pulse rate variability (PRV) is an analysis method to study the physiological conditions of the human body that these small changes can reflect. Studies have shown that using photoplethysmography (PPG) signals to analyze PRV in healthy people can replace HRV to reflect changes in the autonomic nervous system (ANS), and there is a good correlation between the two (21). Further studies have confirmed that PRV in SDB patients also has a good correlation with HRV, thus shifting SDB detection research from ECG signals to PPG signals that are easier to obtain (22–26). Amir et al. (27) conducted PSG synchronous PPG monitoring on 74 volunteers and found that the use of PPG signals to determine sleep breathing status indicated that PPG and PSG have a good consistency and can be used for clinical sleep breathing monitoring.

The Beidou satellite navigation system provides positioning, navigation, and timing services (28), which are divided into open service (29) and authorized service (30). Licensing services: in addition to free and open services to the world, there are services that require an authorization, which is divided into different levels and is distinguished between military and civilian use (31). The Beidou satellite navigation system is very important when calling for help and position when in distress at sea. In view of this situation, IMO has developed a set of global maritime distress and safety systems (GMDSS) (32). The systems consist of three major parts: INMARSAT (Inmarsat system) (33) and COS-PAS-SARSAT (Polar-orbiting Satellite Search and Rescue System) (34), ground-based radio communication system (i.e., Coast Station) (35), and Maritime Safety Information dissemination system (36).

The above research proves the feasibility of using machine learning to study the assistance positioning system for OSA patients. However, most of the above studies are based on small sample data sets and lack large-scale clinical applications. In addition, too many features may be selected in the feature engineering, resulting in too many calculations. Or there are too few functions selected, resulting in low classification accuracy. In addition, when selecting a deep learning classification method, manually setting parameters is too inefficient. At the same time, it may overtrain and lead to test overfitting and, because most research methods are only based on small data sets, in practical applications, the generalization ability of this model is low. Therefore, this topic aims to use machine learning algorithms on the cloud platform to standardize rescue positioning data from different devices.

## Method and Simulation Results

**Figure 1:**
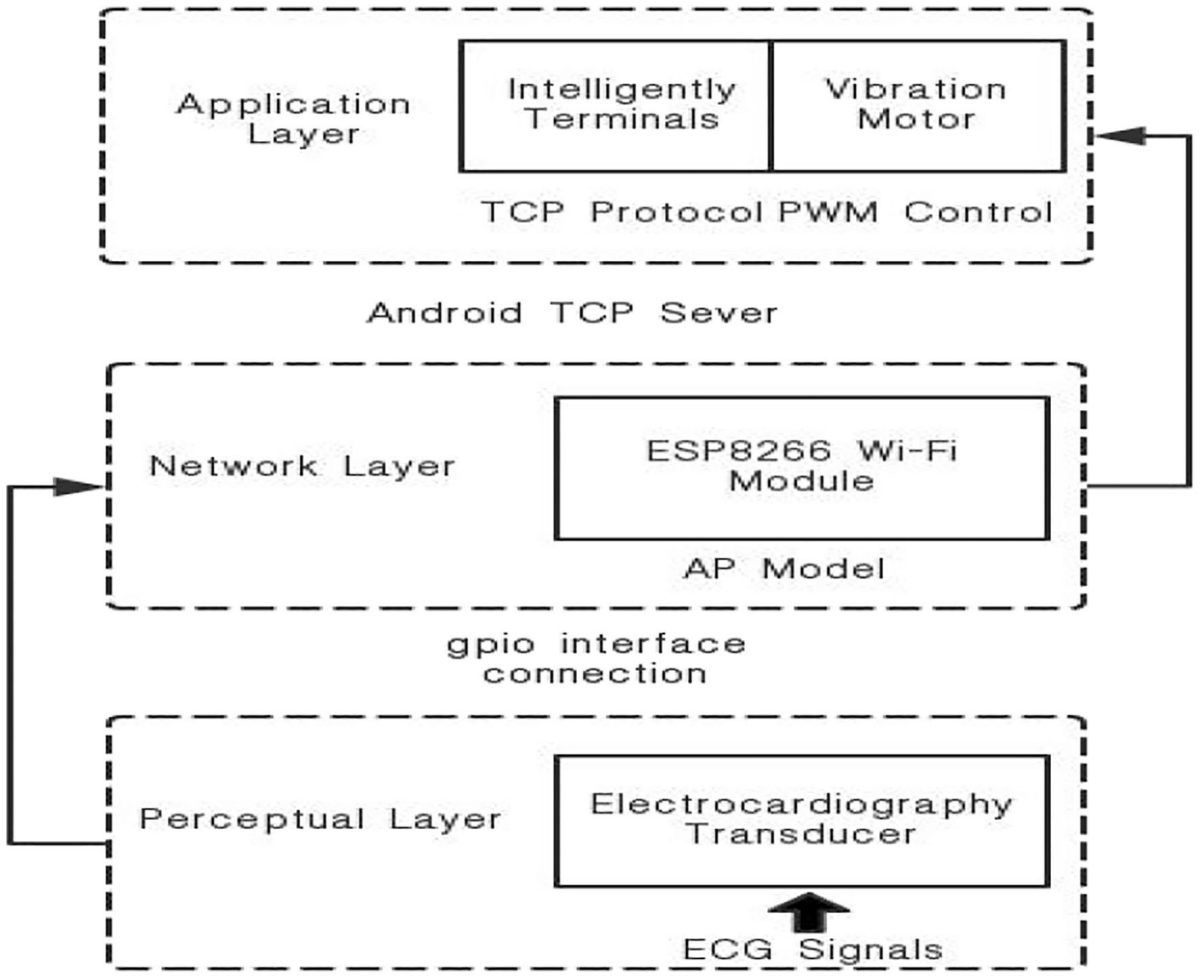
OSA patient monitoring system structure.

The hardware component of the IOT-based sleep disorder monitoring system consists of three parts. The bottom layer is the hardware terminal of the indicator mark, the middle layer is the transmission medium, and the upper layer is the application platform. The beacon terminal includes an ARM main control board, a 4th generation mobile communication module, a Beidou communication module, a Bluetooth communication module, an antenna interface module, and a power management module. In addition, the sides of the housing are provided. There are data transmission interfaces, SOS buttons, and charging ports. There are several sealing rings and fixing screws at the bottom of the housing. However, when a sleep disorder patient is working outdoors, when the drowsiness state occurs, the SOS button can be used to send the help information to the Beidou satellite. Through the auxiliary ground-based augmentation system, Beidou Satellite sends the current status and position coordinates of the sleep-disabled patients to the Beidou rescue platform. The information released by the rescue platform enables the rescue team to quickly and accurately find the location to be rescued. As shown in Figure 1.

### Design of the Perceptual Layer

#### Hardware Design

The whole system is based on ARM main control board, 4th generation mobile communication module, Beidou communication module, Bluetooth communication module, antenna interface module and power management module. In addition, the sides of the housing are provided. There are data transmission interfaces, SOS buttons and charging ports. There are several sealing rings and fixing screws at the bottom of the housing. However, when a sleep disorder patient is working outdoors, when the drowsiness state occurs, the SOS button can be used to send the help information to the Beidou satellite. The Beidou second-generation passive positioning and Beidou active short message communication are combined. The location information of OSA patients can be obtained through the Beidou module, while OSA patients can send disease information through Android mobile phone software operation. The emergency call function is provided in an emergency, and the OSA patient can send his own location information and simple condition information, thereby realizing the emergency rescue function of the OSA patient monitoring system based on the IOT framework.

#### Function Design of Obtaining Position Information

The STM32 development board communicates with the Beidou module by serial port. It uses the command of RNSS format and uses BDGGA to get its own location information and stores it in the cache. When a mobile phone is connected with the terminal through Bluetooth, the data is forwarded to the mobile phone if the mobile phone makes a data request. The mobile phone is transformed into latitude and longitude coordinates through the received Beidou position information and displayed.

### Design of the Network Layer

#### Functional Design of the Mobile Phone

OSA patients can use the Android phone to send AT commands to initialize the Beidou indicator, and obtain their own location information through BLE communication. The latest rescue information and the latest location information obtained through the refresh function. The OSA can also provide a special message input function through the Beidou indicator, input the condition information through the mobile phone and forward it to the terminal, and then the condition message and location information can be sent to the Beidou rescue platform by the Beidou indicator.

#### Design of Communication Protocol Between End and Terminal of Mobile Phone

It is very necessary for the mobile terminal to communicate with the terminal using Bluetooth to maintain complete communication. The mobile phone terminal obtains whether the communication is normal by sending instructions, and the terminal receives the correct return value by continuously sending test information, and providing an LED flicker function display.

#### Terminal Automatic Help Function

By setting the timer, the function of obtaining the position can be used automatically, and the acquired position information can be sent periodically. The B4 function can be started by triggering the button B3 automatic function to stop the automatic function.

The main consideration is to obtain location information, and how to send information for help and other aspects. In the event of a problem, the rescue party can get the message in time, and the suitor can also get the necessary information anytime and anywhere. The interactive function of the display object is mainly realized by Bluetooth module and STM32 main control board.

### Software Design

#### Terminal Part System Design

**Figure 2:**
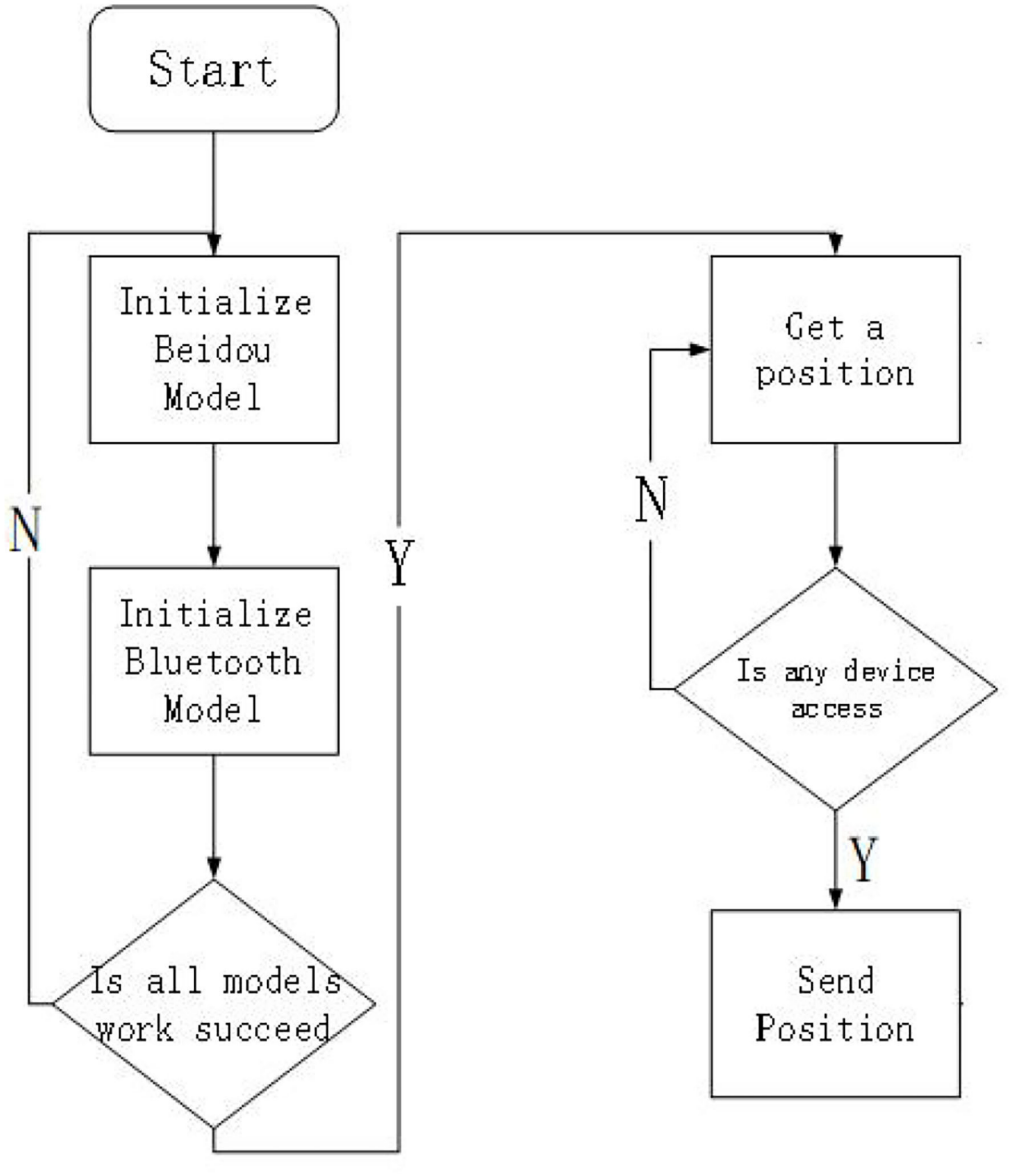
Beidou terminal system design diagram.

Figure 2 illustrates the initialization process of the Beidou indicator, which provides a simple led indicator during normal operation. When you are able to communicate properly, you can view location information from your phone software.

#### Android Client Design

**Figure 3:**
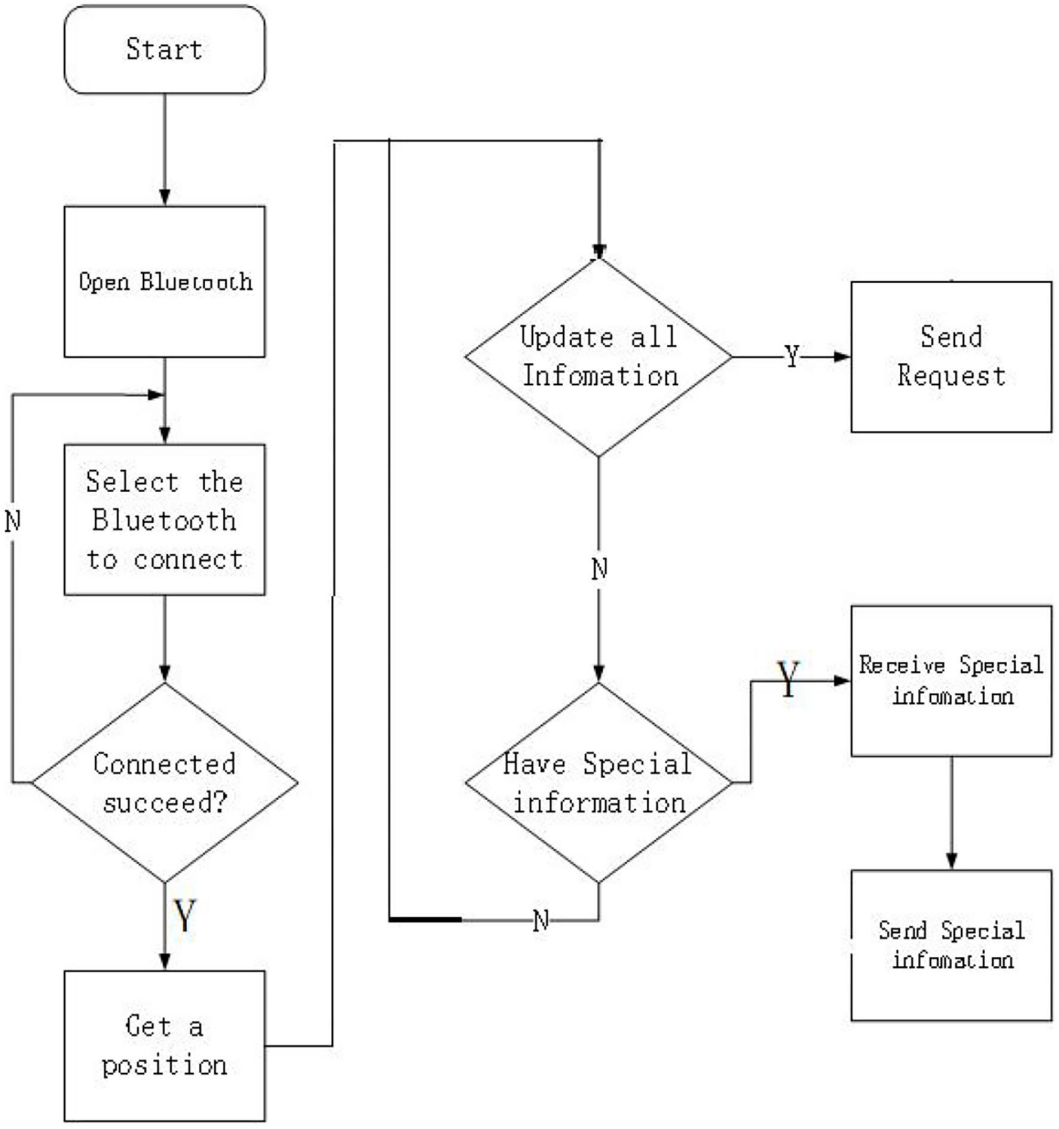
Android client system design.

Figure 3 illustrates the initialization process for the Android phone side of OSA patients. The Beidou indicator mark sends a simple condition message to the mobile terminal, and presses the button SOS on the Beidou terminal to immediately send the condition information to the fixed number already stored in the Beidou indicator. The short message content includes Beidou location information and simple disease information. The Beidou indicator uses the Beidou module to send information. OSA patients can use 4G mobile phones to update data and display location and SMS status information for OSA patients.

### Data Transmission

#### The Server Reception Broadcast Process

**Figure 4:**
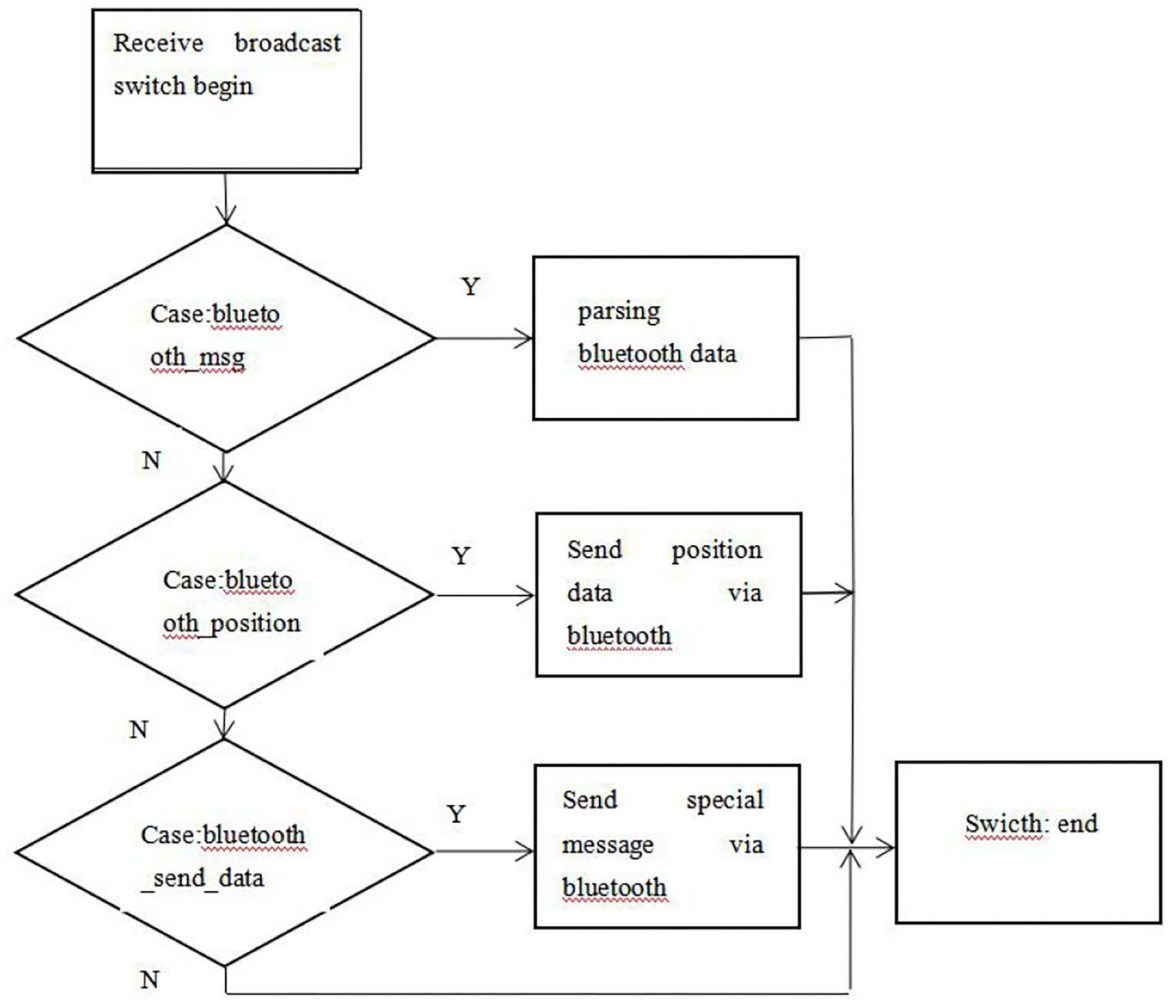
Server receiving broadcast flow chart.

The default encoding mode for Android phones is utf-8, which is used for serial communication to receive and send gdb32 encoded Chinese data. When you need to convert an object to a String object, the encoding system may produce inconsistencies, so the server also encodes and converts characters when performing transceiver control. The Server still does not stop when the Activity application is paused, so receiving information should not stop when the application is not in use at this time. Activity needs to bind to Server (Figure 4).

**Figure 5:**
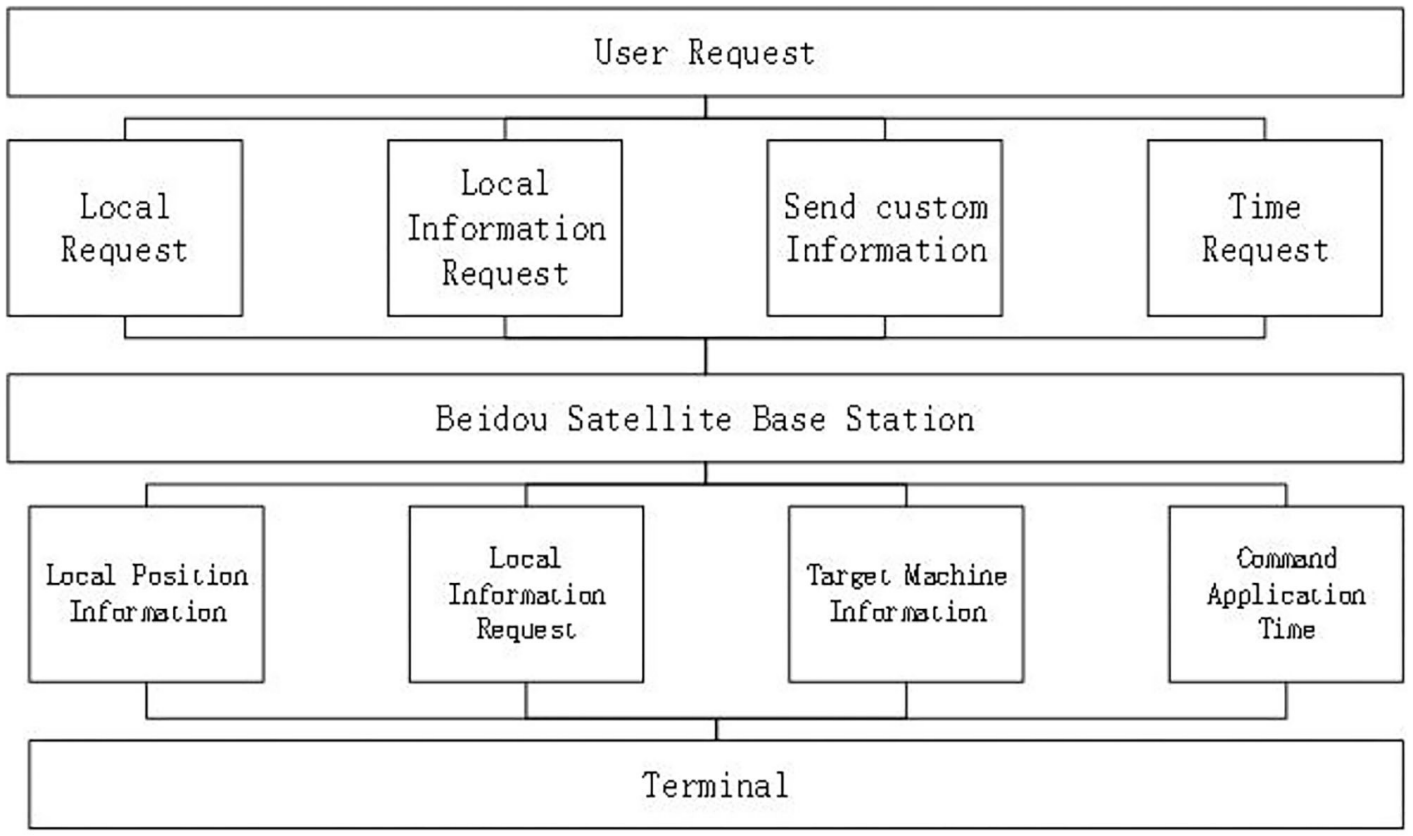
Beidou terminal function service flow chart.

Terminal function service flow, as shown in Figure 5.

## Experimental Study

### Implementation Platform

**Figure 6:**
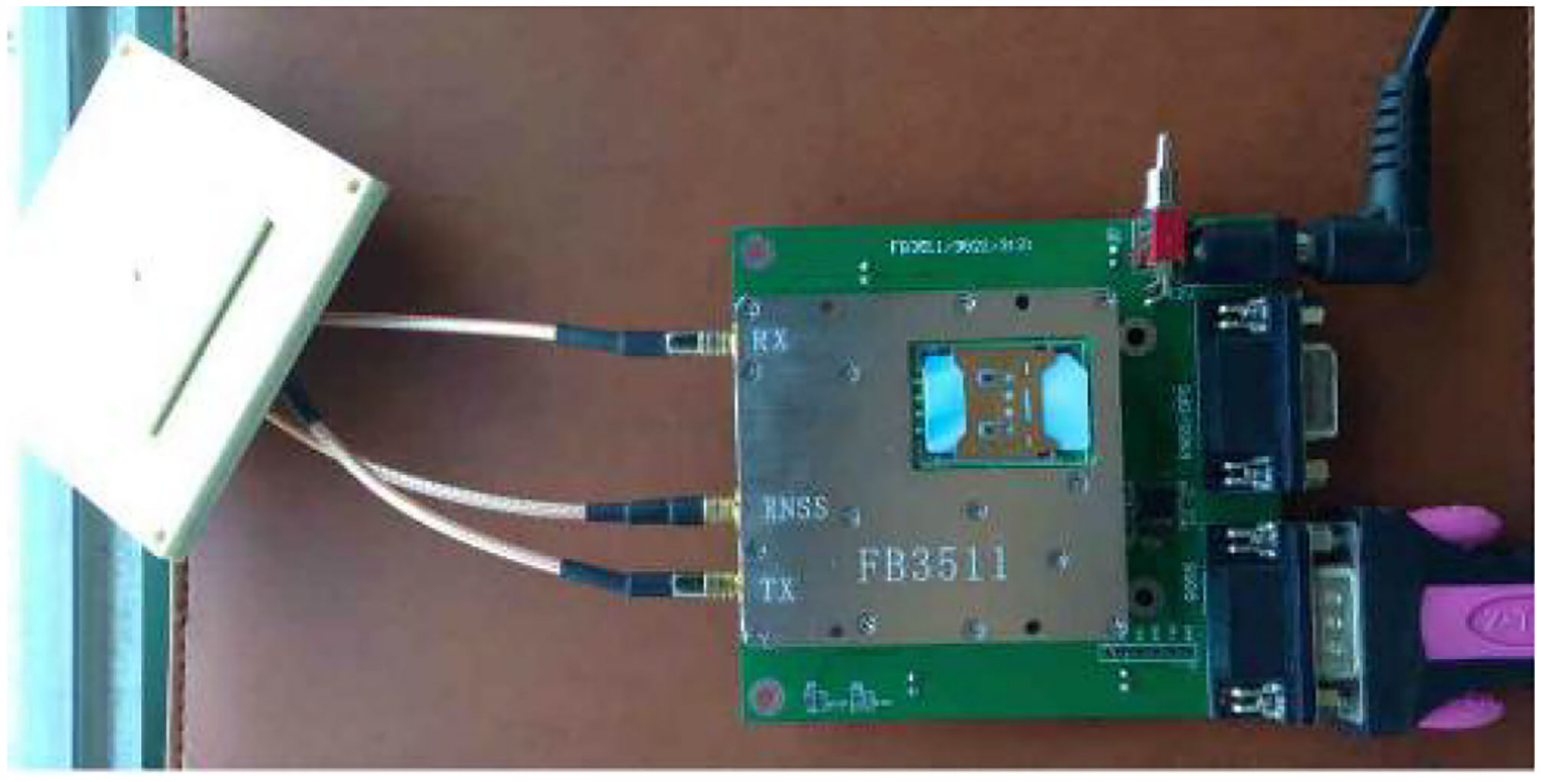
Beidou interactive location-indicating machine.

The Beidou indicator uses the STM32F103RBT chip development board. The code running on the beacon is compiled on the mdk4.9 platform and downloaded using the CooCox development tool. Android phones use android stdio as the development platform for the Android sdk version. The entire mark is shown in Figure 6.

### Android Client

The Beidou indicator uses a switch control to control the switch of the Bluetooth module. If Bluetooth is turned on, you can choose to connect to the Beidou.

Send success messages and location for success as shown in Table 1.

**Table 1:** Success effect of Beidou sending information.

	**Time**	**Longitude**	**Latitude**
T1	12:32:39	119°46'46.6”	46°46'37.6”
T2	12:34:37	119°46'45.9”	46°46'36.9”
T3	12:36:38	119°46'45.8”	46°46'37.2”
T4	12:38:38	119°46'46.3”	46°46'37.3”
T5	12:40:39	119°46'46.1”	46°46'37.1”
T6	12:42:38	119°46'46.6”	46°46'37.5”
T7	12:44:39	119°46'46.6”	46°46'37.3”
T8	12:46:39	119°46'46.5”	46°46'37.2”
T9	12:48:39	119°46'46.4”	46°46'37.3”
T10	12:50:39	119°46'46.5”	46°46'37.3”

Logistic regression is a statistical tool based technique to deal with machine learning problems. We evaluated the results using logistic regression. Logistic regression works with sigmoid function. the accuracy of the LR model is very high (90%). In particular, LR models using all 10 features show better accuracy than other existing models using RQA functionality with an accuracy rate of 90%.

Most importantly, models using LR are still the best in terms of prediction accuracy, precision, recall, and F-measure.

**Figure 7:**
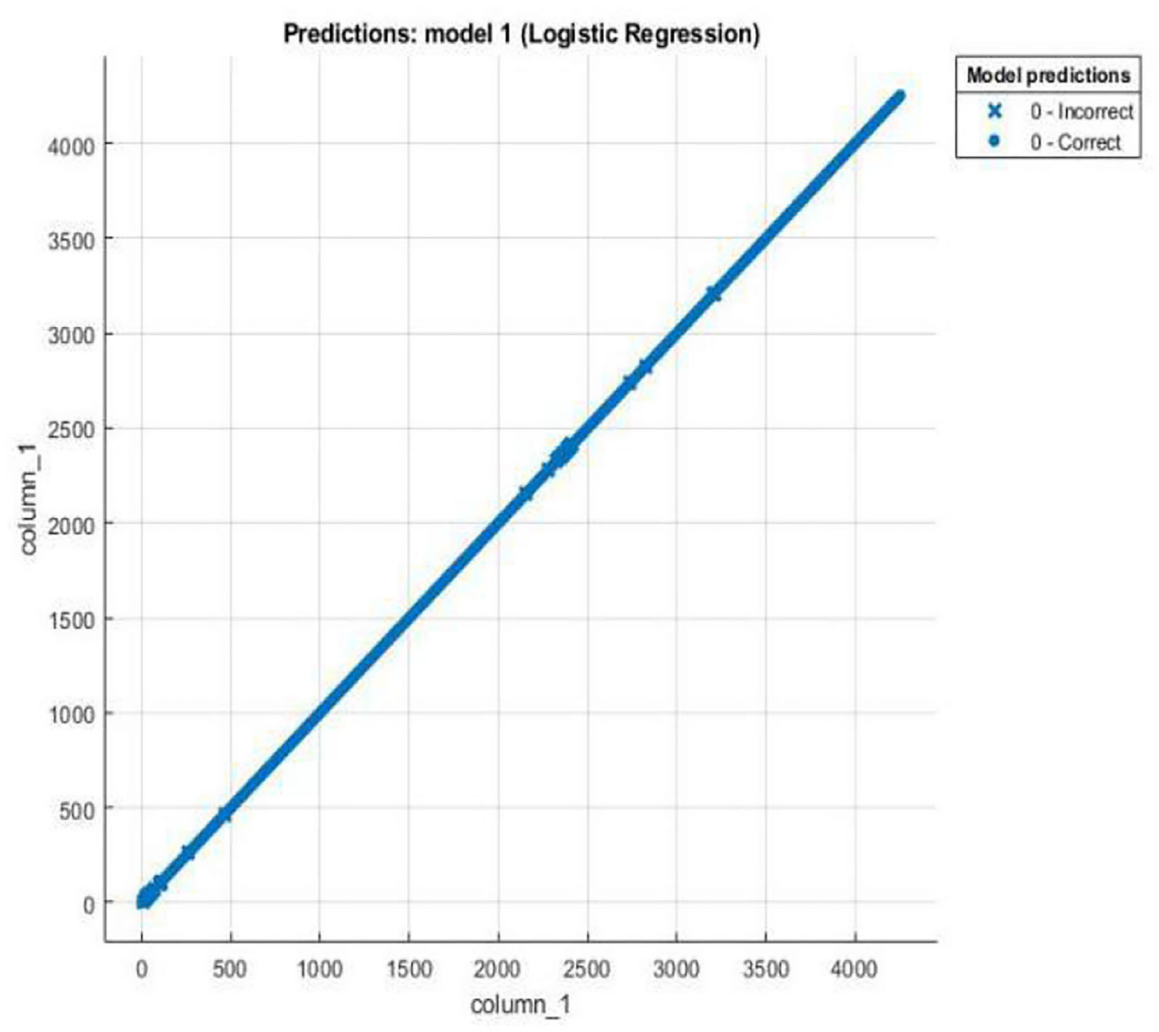
Scatter plot from logistic regression.

**Figure 8:**
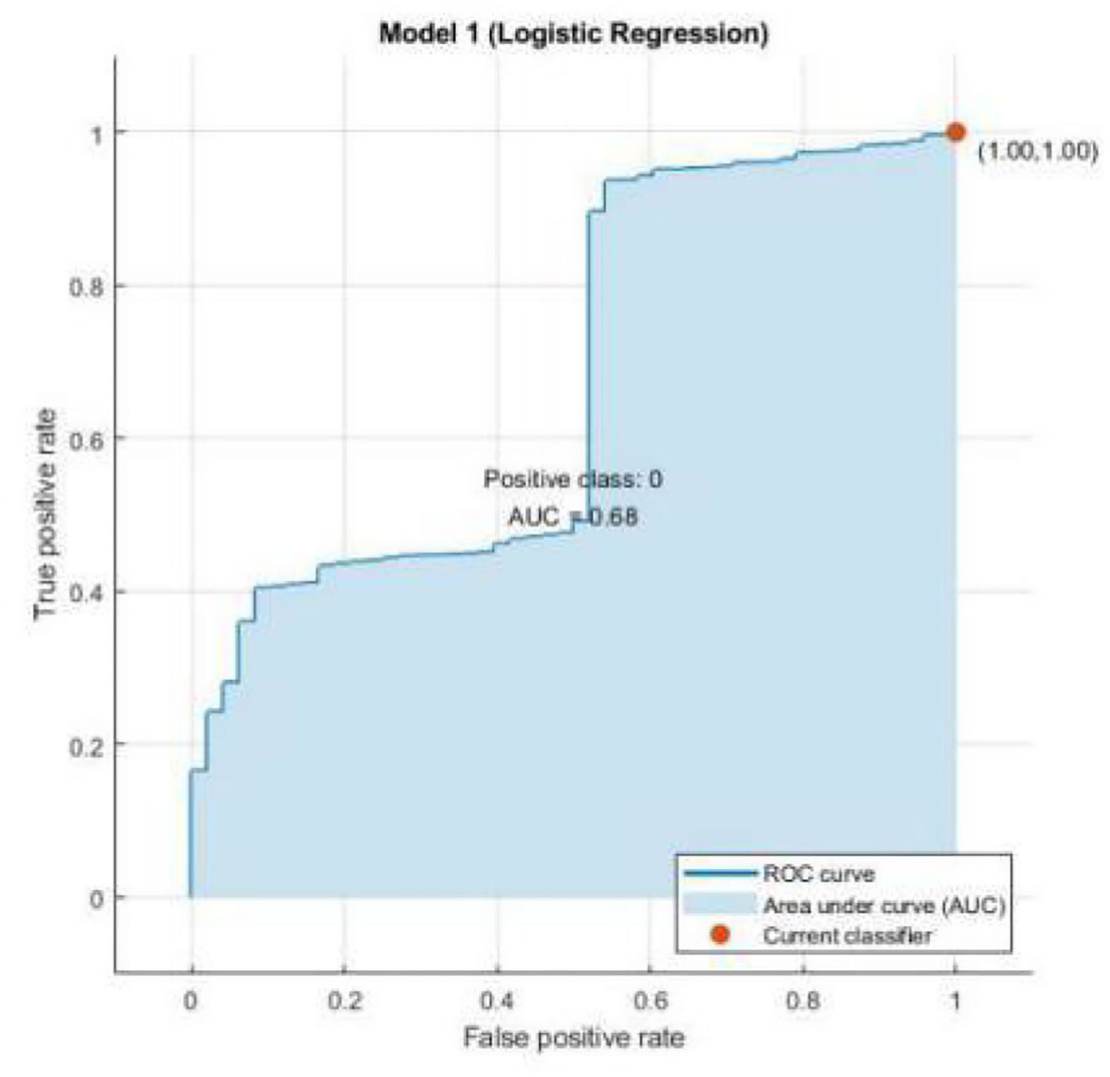
ROC curve from logistic regression.

**Figure 9:**
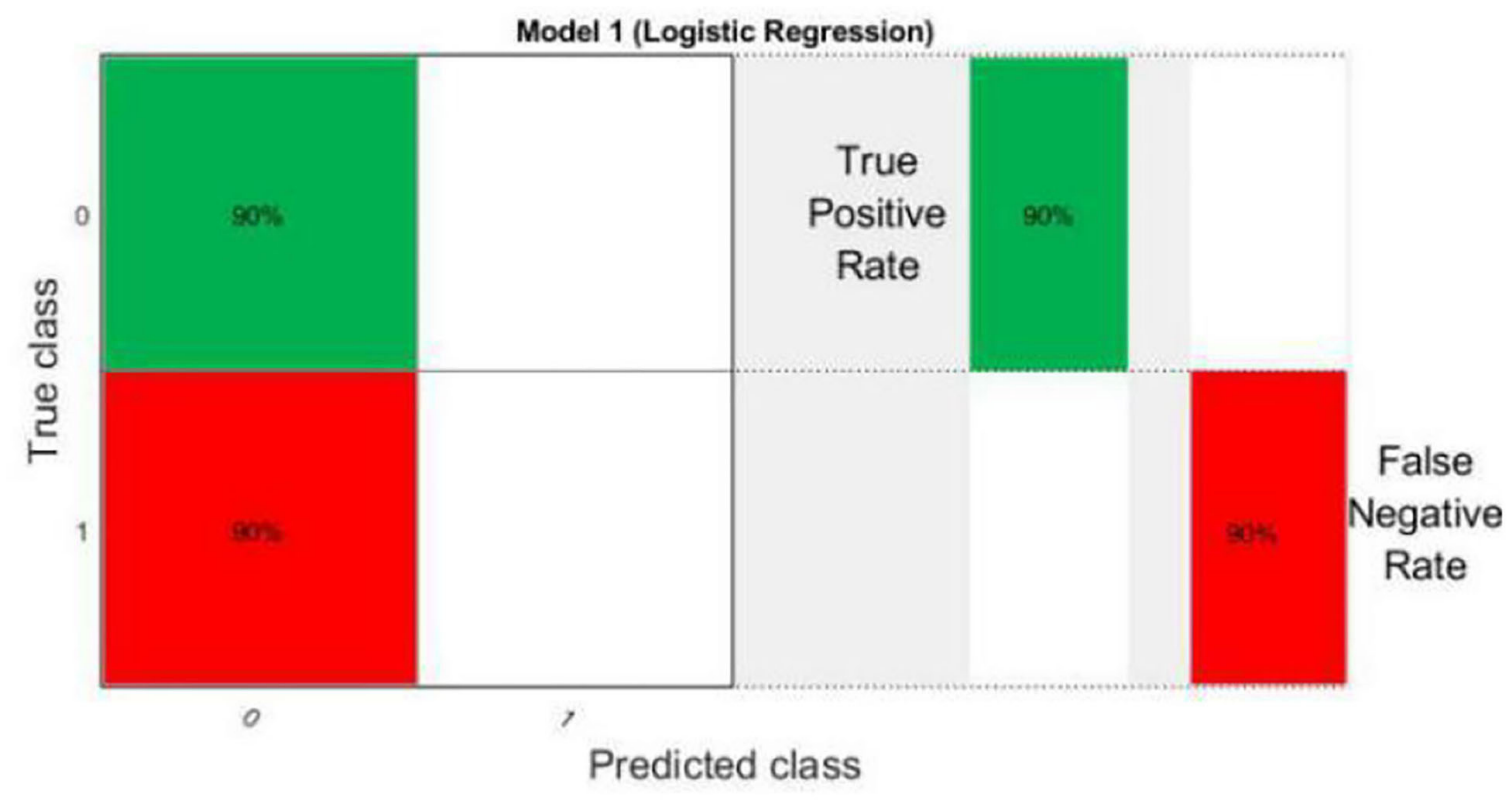
Confusion matrix plot from logistic regression.

**Figure 10:**
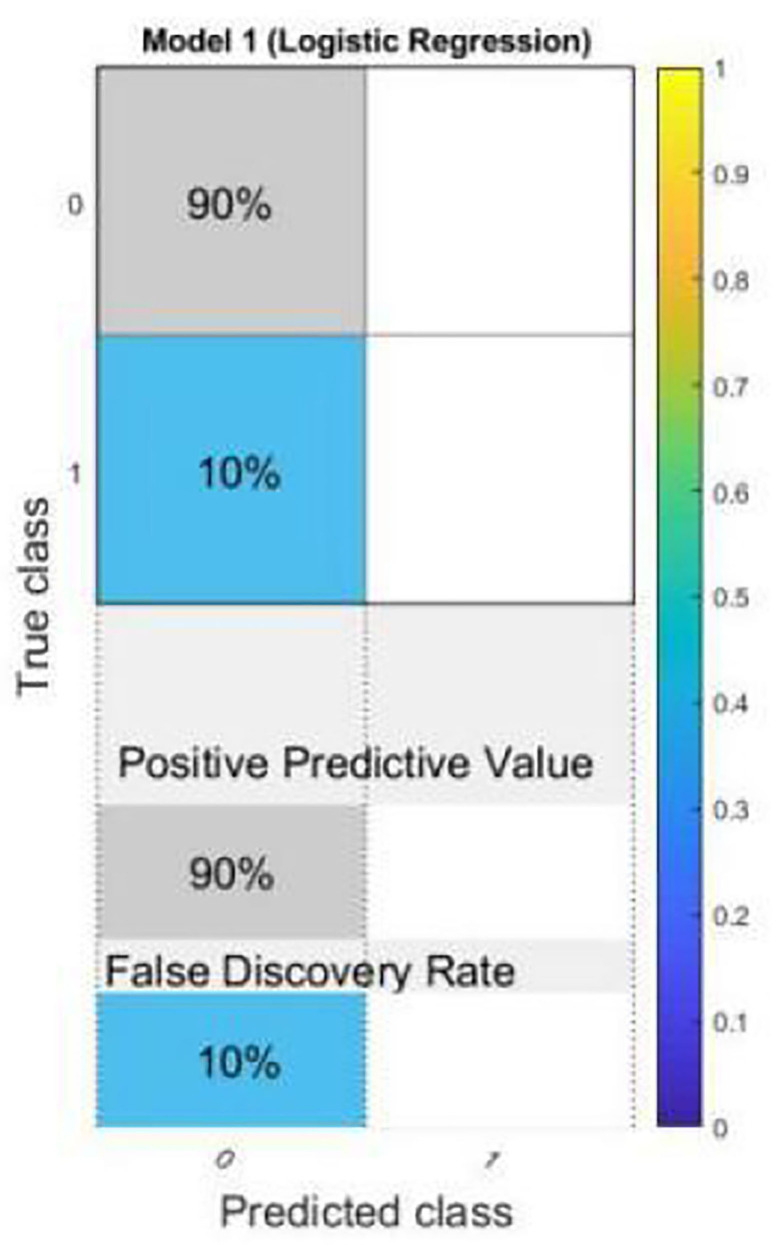
Confusion matrix plot with positive predictive value and false discovery rate from logistic regression.

**Figure 11:**
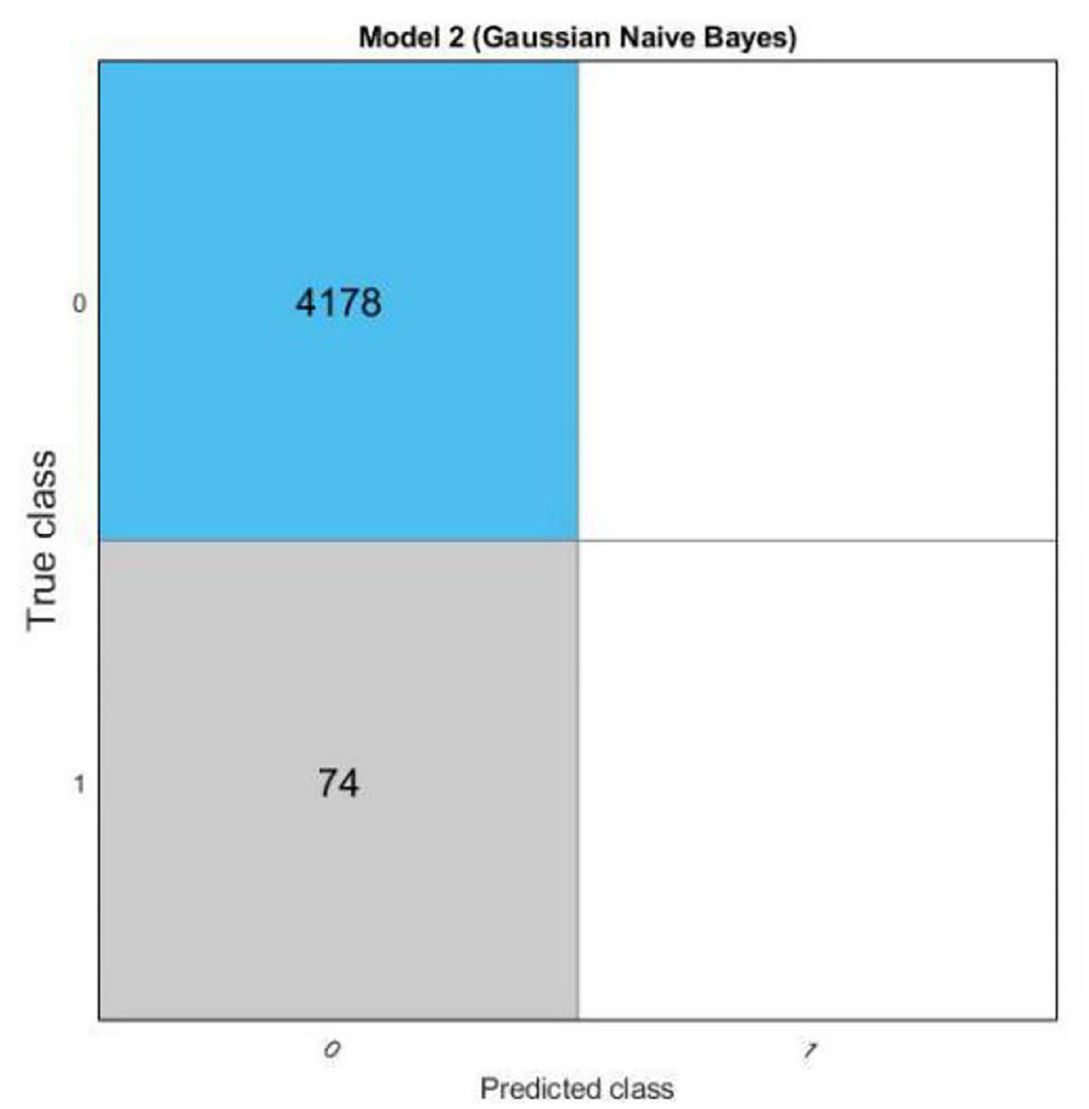
Confusion matrix plot with true class and predicted class from logistic regression.

The results of scatter plot from logistic regression is shown in Figure 7. It is very clear from the figure that logistic regression has performed efficiently to solve the issue of OSA patient monitoring. The ROC curve is shown in Figure 8. Parallel co-ordinate plot also shows that the prediction is accurate. From the confusion matrix in Figure 9, it can be seen that the accuracy is 90%. Moreover, the figure also shows the true positive rate and false negative rate. The Figure 10 shows that positive predictive value is 90 % and false discovery rate is 10%. Figure 11 consists of true class and predicted class.

**Figure 12:**
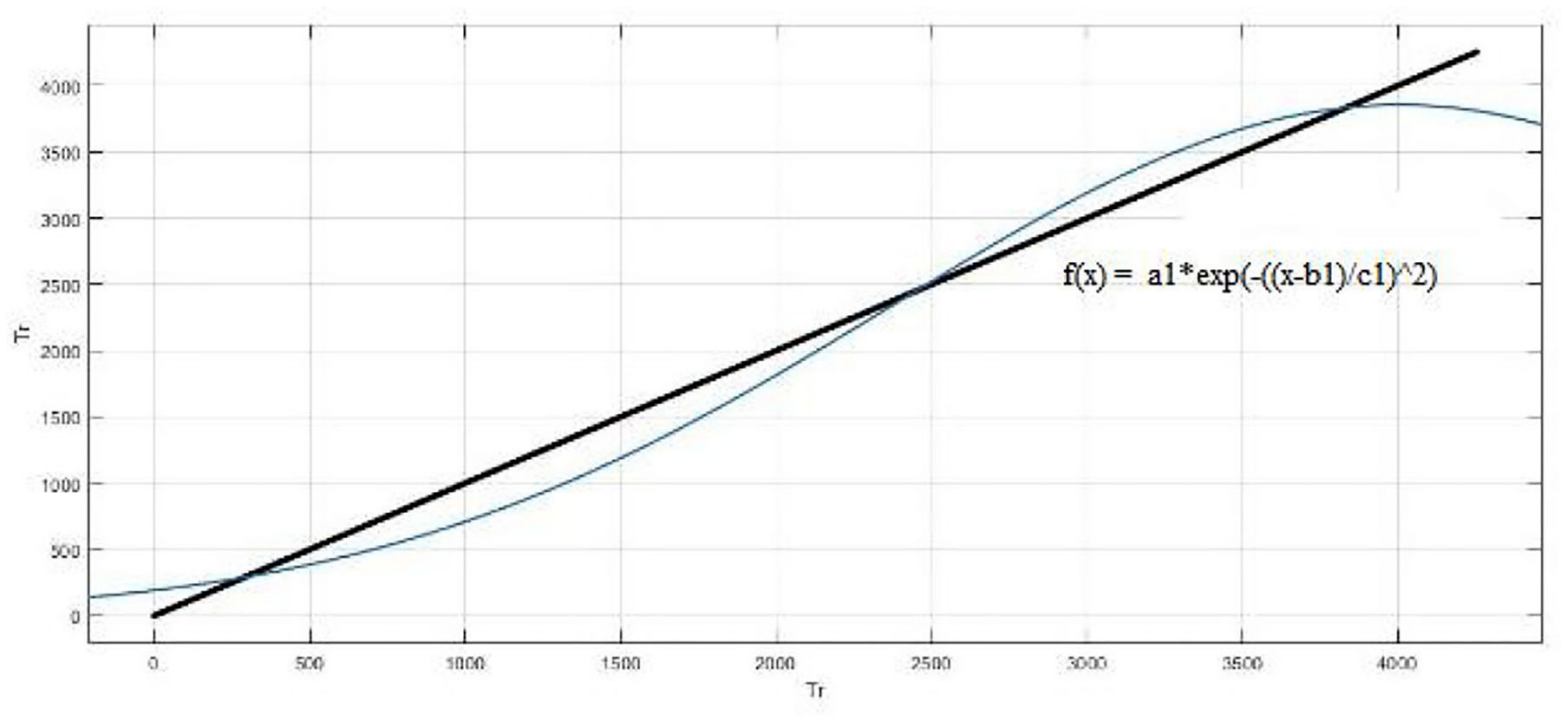
Customized fitting model with *f* (x) = a*[sin(x−pi)] + b*[(x−10)^2^] + c.

A customized fitting model is shown in Figure 12. The coefficients of the model are a = 6.989, b = 0.0002727 and c = 247.1 (237.6, 256.7). The R-square value: 0.9222 and root mean squared error is 382.8.

**Figure 13:**
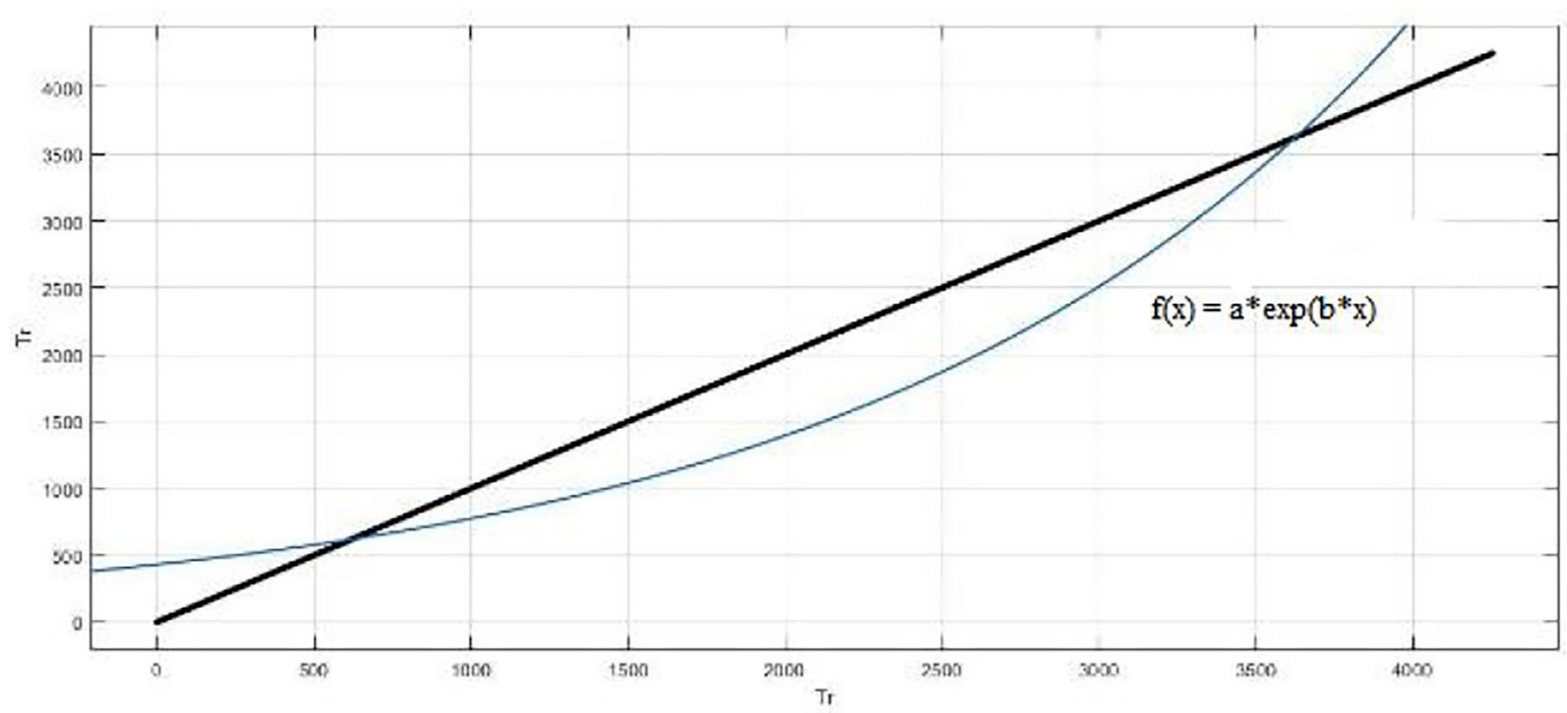
Exponential fitting model.

**Figure 14:**
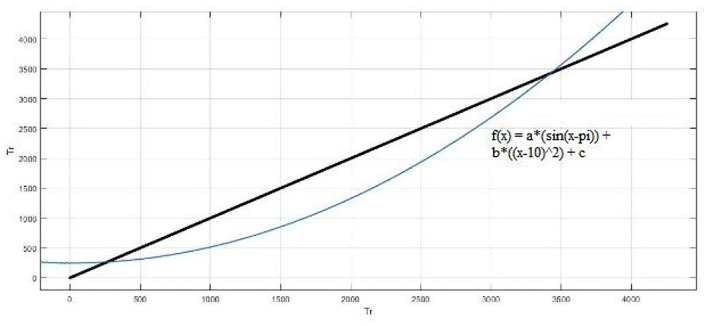
Gauss fitting model.

Figure 13 shows that the result is also fitted with exponential fitting [f(x) = a^*^exp(b ^*^ x)]. The coefficients of the model are a = 430.5, and b = 0.0005877. The R-square value: 0.8975 and root mean squared error is 439.5. The Gauss fitting model is shown in Figure 14. The equation of gauss fitting model is *f* (x) = a1^*^exp[−((x–b1)/c1)^2^]. The coefficients of the model are a1 = 3855, b1 = 4005, and c1 = 2310. The R-square value: 0.9795 and root mean squared error is 196.6.

The motivation of the current work is to locate and rescue patients with OSA. Whether the positioning is accurate or not is actually a problem of binary classification of rescue targets. We use logistic regression methods to create classification models. The test result data set is divided into training and test data. Among them, 70% of the data is used for training the model, and 30% of the data is used for the test results. The simulation results confirmed that logistic regression can be used to classify the rescue positioning results of patients with sleep disorders. The positioning accuracy of this system is 90%.

## Conclusions

The OSA sleep disorder patient monitoring system is based on the IoT framework structure. The entire system provides real-time disease reporting information, precise location information, and physician assistance information for OSA patients. The terminal hardware is realized by the Beidou indicator module, the 4G mobile communication module, and the BLE communication module. Through the secondary development of the Android mobile phone SDK development kit, it is convenient for users to operate. The special operation of shielding the Beidou indicator is made to make this OSA patient monitoring and rescue system easier to integrate into the existing hospital emergency system. The system currently only provides the Android mobile phone client as the input and receiving end of the message, which is convenient for the user, who is able to interact with the doctor in real time through the smart phone. The OSA monitoring system has self-healing capabilities and is extremely robust, providing system reliability. This system test shows that the OSA patient system we developed has lower power consumption and a simpler hardware composition. In addition, our OSA patient monitoring and rescue system has a stable and simple operation while meeting design requirements. In short, our system is an innovative application of IoT technology in modern medicine, with a particular focus on the effective management of OSA patients. In the future, we will explore how to further improve system performance by integrating more sensors to monitor OSA patients in a more comprehensive manner.

The simulation results of this research show that the use of machine learning techniques (such as logistic regression) is very suitable for building a classifier for the accuracy of the rescue positioning of patients with sleep disorders. LR has the best performance in all aspects of prediction accuracy, accuracy, recall, and prediction.

These high-accuracy results will encourage researchers to more rigorously study the accuracy of sleep disorder rescue positioning data, and use logistic regression for binary output in more practical classification problems. In the next phase or research, we plan to use deep learning, convolutional neural networks, and transfer the learning to the classification of sleep disorder rescue positioning accuracy. In addition, it aims to collect data from hospitals and use machine learning algorithms to evaluate and predict the accuracy of sleep disorder rescue positioning. It is possible to manufacture devices with IOT to monitor sleep abnormalities in real time.

## Data Availability Statement

The original contributions presented in the study are included in the article/supplementary material, further inquiries can be directed to the corresponding author/s.

## Author Contributions

CL, CX, and DM conceived the study, designed the experiments, analyzed the data, and wrote the whole manuscript. MB and LM provided the preprocessed data. ZZ, LS, and RY carried out experiments. HQ helped to analyze the data and experiments result. YS revised the manuscript.

## Funding

This work was supported by the Fujian Provincial Scholarship Council, the National Science Foundation of China (No. 61773124), the Fujian Social Science Foundation (FJ2021BF029), the Fujian Natural Science Foundation (2018J01796), the Fujian Science and Technology Department Foundation (2019I1009), Project of Youth Foundation of Fujian Education Department (JAT191104), the Foundation of Fuzhou University Zhicheng College (KJ20190001), and the Foundation of National College Student Innovation and Entrepreneurship Project (ZJ1934). In part by The Open Fund of Provincial Key Laboratory of Eco-Industrial Green Technology-Wuyi College.

## Conflict of Interest

The authors declare that the research was conducted in the absence of any commercial or financial relationships that could be construed as a potential conflict of interest.

## Publisher's Note

All claims expressed in this article are solely those of the authors and do not necessarily represent those of their affiliated organizations, or those of the publisher, the editors and the reviewers. Any product that may be evaluated in this article, or claim that may be made by its manufacturer, is not guaranteed or endorsed by the publisher.
